# Treating social anxiety disorder remotely with cognitive therapy

**DOI:** 10.1017/S1754470X2000032X

**Published:** 2020-07-16

**Authors:** Emma Warnock-Parkes, Jennifer Wild, Graham R. Thew, Alice Kerr, Nick Grey, Richard Stott, Anke Ehlers, David M. Clark

**Affiliations:** 1 Department of Experimental Psychology, University of Oxford, Oxford, UK; 2 Oxford Health NHS Foundation Trust, Oxford, UK; 3 Sussex Partnership NHS Foundation Trust, UK; 4 University of Sussex, UK; 5 King’s College London, London, UK

**Keywords:** CBT, cognitive therapy, COVID-19, distance therapy, remote therapy, social anxiety disorder, social phobia

## Abstract

Remote delivery of evidence-based psychological therapies via video conference has become particularly relevant following the COVID-19 pandemic, and is likely to be an on-going method of treatment delivery post-COVID. Remotely delivered therapy could be of particular benefit for people with social anxiety disorder (SAD), who tend to avoid or delay seeking face-to-face therapy, often due to anxiety about travelling to appointments and meeting mental health professionals in person. Individual cognitive therapy for SAD (CT-SAD), based on the Clark and Wells (1995) model, is a highly effective treatment that is recommended as a first-line intervention in NICE guidance (NICE, 2013). All of the key features of face-to-face CT-SAD (including video feedback, attention training, behavioural experiments and memory-focused techniques) can be adapted for remote delivery. In this paper, we provide guidance for clinicians on how to deliver CT-SAD remotely, and suggest novel ways for therapists and patients to overcome the challenges of carrying out a range of behavioural experiments during remote treatment delivery.

**Key learning aims:**

1. To learn how to deliver all of the core interventions of CT-SAD remotely.
2. To learn novel ways of carrying out behavioural experiments remotely when some in-person social situations might not be possible.

## Introduction

The COVID-19 pandemic has forced clinicians to adapt their clinical practice. Therapies that were normally delivered in a face-to-face clinic-based format have had to be delivered remotely via video-conferencing. This forced adaptation may have a silver lining in the sense that it opens up a new option for treatment delivery that may be popular with some patients in the post-COVID world. Remote delivery may help overcome some specific barriers to help-seeking, such as anxiety about travelling to clinics, the stigma of being seen in a mental health unit, and the cost and time of travelling to appointments. For patients with social anxiety there may be the added appeal of not having to start therapy with an in-person meeting in a strange environment.

Social anxiety disorder is one of the few common mental health disorders for which the National Institute for Health and Care Excellence (NICE) does not recommend stepped care but instead recommends that patients are immediately offered face-to-face high-intensity therapy. Cognitive therapy for SAD (CT-SAD) based on the Clark and Wells (1995) model is one of two high-intensity cognitive behavioural therapies that NICE (2013) recommends as first-line treatments for the disorder. This recommendation is based on a series of clinical trials conducted in the UK, Sweden, Norway, Germany and Japan, which have shown that CT-SAD is superior to a wide range of other interventions including exposure therapy (Clark *et al*., 2006), group cognitive behaviour therapy (Ingul *et al*., 2014; Mörtberg *et al*., 2007; Stangier *et al*., 2003) interpersonal psychotherapy (Stangier *et al*., 2011), psychodynamic psychotherapy (Leichsenring *et al*., 2013), selective serotonin re-uptake inhibitors (Clark *et al*., 2003; Nordahl *et al*., 2016), medication-based treatment as usual (Mörtberg *et al*., 2007; Yoshinaga *et al*., 2016), pill placebo (Clark *et al*., 2003) and psychological placebo (Ingul *et al*., 2014).

In this paper, we describe how to deliver CT-SAD remotely. First, we describe the cognitive model (Clark and Wells, 1995) that underpins CT-SAD and consider a number of general practical considerations for remote delivery. We then cover each of the core CT-SAD interventions (see Table 1) and describe how to adapt them when working remotely. Videos demonstrating how to implement all the core interventions outlined in this paper are freely available on the Oxford Centre for Anxiety Disorders and Trauma (OxCADAT) website: www.oxcadatresources.com. The website also includes Word copies of all the recording sheets and self-report questionnaires referred to in this paper. Therapists are welcome to download and use these with their patients.

**Table 1.** Core components of remotely delivered CT-SAD outlined in this paper

*CT-SAD treatment components*	
Treatment components typically used with all patients:	
1.	Collaboratively developing a personalised cognitive model of their social anxiety
2.	An experiential exercise to demonstrate the adverse effects of self-focused attention and safety behaviours (the ‘self-focused attention and safety behaviours experiment’)
3.	Video and still photograph feedback to correct negative self-imagery
4.	Attention training to practise focusing externally
5.	Behavioural experiments to test negative beliefs by dropping safety behaviours and focusing attention externally in social situations or purposefully displaying feared behaviours or signs of anxiety (decatastrophising)
6.	Developing a therapy blueprint
Treatment components used as required:	
7.	Surveys to loosen beliefs alongside behavioural experiments
8.	Using virtual audiences to gain confidence in public speaking and test specific beliefs
9.	Addressing anticipatory worry and post-event rumination
10.	Memory work (discrimination training and memory re-scripting) to reduce the impact of early socially traumatic experiences
11.	Additional techniques to address persistent unconditional beliefs and self-criticism

## The cognitive model of SAD (Clark and Wells, 1995)

CT-SAD is based on the Clark and Wells (1995) model, which proposes that patients with SAD are vulnerable to becoming anxious in social situations because they have developed negative assumptions about themselves and their social world. These include excessively high standards for social performance (e.g. ‘I must always be interesting’), conditional beliefs about the consequences of behaving in a particular way (e.g. ‘If I show anxiety people will think I am incapable’) or unconditional negative beliefs about themselves (e.g. ‘I am boring’, ‘I am unlikable’, ‘I am inadequate’). Activation of these assumptions in social situations generates anxiety because it leads patients to predict that they will come across badly (e.g. ‘I won’t have anything to say’, ‘I will shake’, ‘I will blush’, etc.) and to negatively evaluate their performance (‘I am being boring’). These negative thoughts are often accompanied, and reinforced, by negative and distorted self-images and impressions, for example imagining that one looks beetroot red when mildly blushing. The images are often based on anxious feelings (hot face = beetroot red appearance; feeling shaky means I am visibly shaking). However, they can also relate to past social traumas (such as a sense of the self as being boring and rejected, linked to bullying experiences at school).

Two key processes tend to maintain social anxiety disorder by preventing patients from discovering that their negative thoughts are unrealistic. First, in social situations patients become excessively self-focused. Instead of predominantly being focused on the social interaction, patients tend to shift to an internal focus of attention, monitoring how they feel and how they think they appear to others. This internal focus makes them more aware of internal information (anxious feelings and images) that reinforces their negative thoughts and prevents them from picking up any positive responses from others. The second is use of safety behaviours that are carried out with the intention of preventing feared outcomes (e.g. preparing things to talk about if the person fears not having anything to say; hiding face if patient is worried about blushing, etc.) but paradoxically prevent patients from discovering that their fears are excessive. An interesting feature of safety behaviours is that they can also have a negative effect on the behaviour of other people. For example, someone who is worried that she is uninteresting may constantly monitor what she is saying and censor some utterances. This may make her appear to others as if her ‘mind is somewhere else’ and she is not interested in them or the conversation. As a consequence they may be less welcoming, apparently reinforcing her fears. Finally, patients with social anxiety are also habitually self-critical and can excessively ruminate on perceived social failings, which further reinforces patients’ negative beliefs and self-impressions.

### General points about remotely delivered CT-SAD

#### We recommend that conducting all sessions via video conference is the best way to ensure that the key components of CT-SAD are delivered effectively

Some interventions are possible over the telephone, but a number of core interventions are not (for example in-session behavioural experiments meeting other people and video feedback). In addition, we would advise against starting treatment with telephone delivery as this may inadvertently encourage avoidance of showing oneself to others.

#### CT-SAD is typically delivered in up to 14 weekly therapy sessions over a period of 3–4 months

For face-to-face therapy, it is recommended that therapy sessions are up to 90 minutes long to ensure that therapists and patients can conduct behavioural experiments (both in the office and outside) during the sessions and have sufficient time to discuss the results of the experiments. In remotely delivered therapy, we would strongly recommend that therapists also aim to conduct in-session behavioural experiments in many treatment sessions. When this happens, an extended session would be helpful.

#### Practical issues for patients

It can be useful to discuss a number of practical issues at the start of treatment, including:

##### Patients may have concerns about privacy when having sessions in their own home

This can be particularly problematic for people with SAD, who often feel self-conscious talking about personal issues. This can usually be addressed by problem-solving with the patient to find the most private location in or outside their home. Using a set of headphones/ear buds for the sessions is also helpful.

##### Technical set-up

Before starting therapy, it is recommended that therapists have a test video call with their patients to check that the chosen video call programme works on the patient’s computer and with their internet connection. Patients may need to adjust where they sit in order to ensure that their camera captures them clearly with a well-lit image (e.g. avoid sitting with a bright window behind you). It can also be useful to agree that the therapist will contact the patient by telephone should connection issues arise during the call.

##### Minimising distractions

When patients are receiving treatment in their own home, there are many possible events (childcare, telephone calls, etc.) that can interrupt a session. It is important to discuss with patients about how these interruptions can be minimised. For example, turning off telephones during therapy sessions, identifying a quiet place in the house, etc.

##### Screen sharing

In face-to-face CT-SAD, therapists and patients work collaboratively, often making notes together on a white board or using paper and pen (for example when drawing out the cognitive model, setting up and discussing behavioural experiments, comparing ratings made before and after viewing video footage of experiments, etc.). Many of the documents that therapists and patients might complete together in face-to-face sessions are available at the OxCADAT resources website. In remotely delivered therapy, blank copies can be emailed to patients to complete in therapy sessions with the therapist following using screen-share (a function available on most video conference platforms) or vice versa. As in face-to-face therapy it is helpful if patients keep a file containing all of the completed documents, either on their computer or printed hard copies.

##### It is recommended that patients hide their self-view in video conference calls

Patients with SAD are inherently self-conscious and self-focused. When using most video conferencing facilities, people often see a video image of themselves on screen, alongside video images of the other people they are speaking to. For patients with SAD, this could increase self-consciousness, interfering with their concentration during the session. Depending on the video conferencing system used, it is often possible for them to turn off the self-view, minimise it, or drag the video image of themselves off screen.

##### CT-SAD sessions should ideally be recorded, with patient consent

A lot of material is covered with CT-SAD sessions. We have found that it is helpful for patients to take an audio recording on their telephone of sessions so they can listen to it afterwards. This helps to maximise learning. In session 3, and some later sessions, video feedback is used to help correct patients’ excessively negative impressions of the way they think they appear. Many video conferencing facilities have in-built recording functions that could be used if this can be done securely, fitting with local service policies. If the service does not permit therapist side recording, some video conferencing facilities will allow the patient to make and store the recordings themselves. However, if the patient records video of the call, they should be instructed to hold off from watching the conversations until they can do so together with the therapist in a video feedback session. When recordings are being made during remote therapy, the therapist should first check that the method of recording will capture both video of the patient and the person they are talking to. This is important for effective video feedback (so that the patient can see both themselves and the reactions of the other person in the interaction).

#### Therapeutic relationship in remote CT-SAD

##### The treatment of people with SAD is complicated by the fact that the therapist is, at least initially, a stranger and so may be seen as a ‘phobic object’

The fears the patient experiences with others may also be experienced with the therapist. This is a complication that therapists need to be aware of and make adjustments for, to establish a good therapeutic relationship. For example, as direct eye contact via the webcam can increase autonomic arousal (Hietanen *et al*., 2020), therapists should remain warm and accepting, but try not to stare straight into the video camera when asking the patient a question in early sessions. Using screen share can help as both of you are looking at a document on screen rather than staring directly at each other (for example, when developing the model).

##### Asking the patient for feedback regularly is especially important when delivering treatment remotely

Patients with SAD can find it difficult to raise concerns or ask questions early in therapy. When delivering treatment remotely, some of the non-verbal cues a therapist looks out for in face-to-face sessions are harder to detect, for example signs of distress or that something the therapist said was misunderstood. This makes asking for feedback regularly especially important when delivering treatment remotely. This is particularly true when using the sharing screen function, as at these times the therapist’s view of the patient is generally smaller.

### Questionnaires to guide therapy

Table 2 lists the main questionnaires that are used in therapy. Patients are encouraged to complete a measure of the severity of their social anxiety symptoms (SPIN or LSAS) every week in order to monitor change. They are also encouraged to regularly complete measures of the processes that are targeted in therapy. Word copies of these measures are available from the OxCADAT resources website and can be emailed to patients to complete and return before a session. The Social Cognitions Questionnaire (weekly administration) identifies patients’ main fearful concerns. Therapy sessions tend to focus on the negative thoughts with the highest belief ratings. The Social Behaviour Questionnaire (pre-, mid- and post-treatment) helps identify safety behaviours that patients will be encouraged to drop as they progress through treatment.

**Table 2.** Measures given during CT-SAD to guide treatment

Measure	Variables measured	Frequency
***Symptom measures***		
Liebowitz Social Anxiety Scale (Liebowitz, 1987)	Outcome measure of severity of social anxiety with a comprehensive assessment of feared situations	Each session
Social Phobia Inventory (Connor *et al*., 2000)	Outcome measure of severity of social anxiety	Each session
***Process measures***		
Social Cognitions Questionnaire (SCQ; Clark, 2005)	Negative cognitions in social situations (e.g. I am blushing) (frequency and belief)	Each session
Social Phobia Weekly Summary Scale (SPWSS; Clark *et al*., 2003)	Avoidance, self-focused attention, anticipatory anxiety, post-event rumination	Each session
Social Behaviours Questionnaire (SBQ; Clark, 2005)	Safety behaviours used in social situations	Start, middle and end of therapy
Social Attitudes Questionnaire (SAQ; Clark, 2005),	Common beliefs about the self that fall into three categories: excessively high standards for social performance, conditional and unconditional beliefs	Start, middle and end of therapy

### Developing goals and an individualised cognitive model

**Figure 1.**
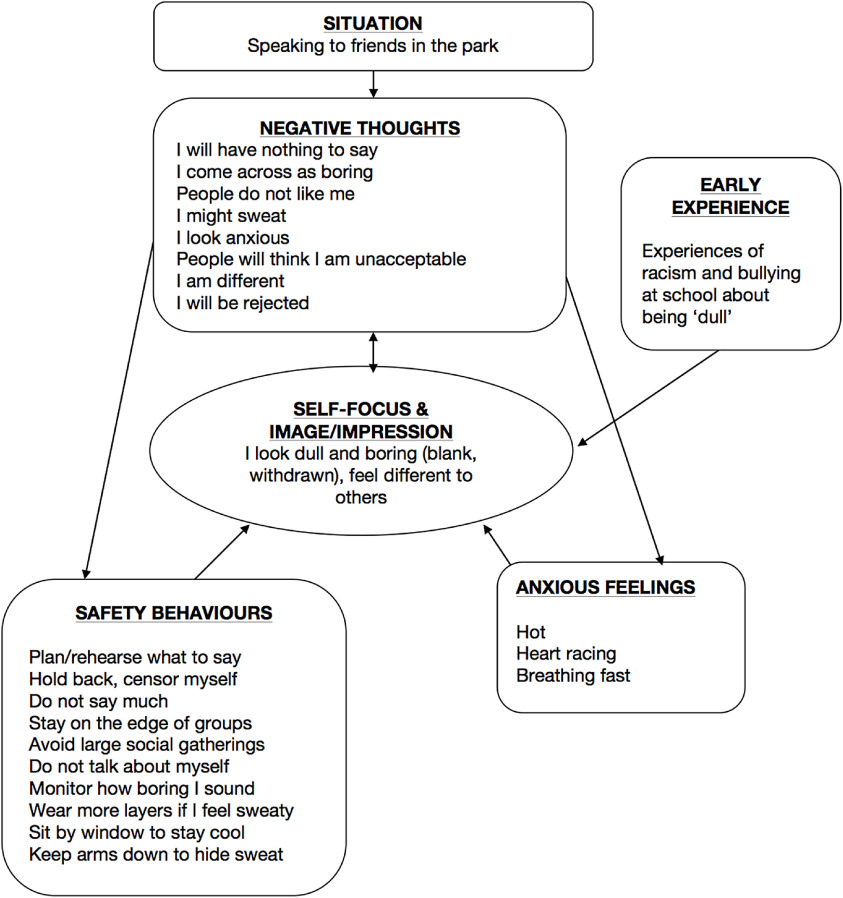
Example of individualised cognitive model drawn out via screen share during the first remotely delivered CT-SAD session.

The first steps in treatment are to help the patient develop specific goals for therapy and to collaboratively develop an individualised version of the cognitive model that will guide therapy, demonstrating how negative thoughts, self-images, focus of attention, safety behaviours and the physical and cognitive effects of anxiety maintain SAD. In face-to-face treatment this would typically be done using a whiteboard in the therapy room. There are a number of possibilities for drawing out the model using screen share via video conferencing. The model could be typed out and developed part-by-part (e.g. adding the boxes and arrows throughout the discussion) using Word or another application. Alternatively, a blank Word version of the model is available to download from the OxCADAT resources website and can be used effectively, if it is explained that it will be individualised to make sense for the person the therapist is working with. Alternatively, a model could be drawn out on paper and a digital copy shared by email. Figure 1 shows an example model drawn out in the first treatment session via screen share.

### Self-focused attention and safety behaviours experiment

In order to discover more about the effects of self-focused attention and safety behaviours on increasing social anxiety, an experiential exercise is typically done in the second session of CT-SAD (see therapist guides and video illustrations on the OxCADAT resources website, for details). The patient takes part in two brief social interactions. The type of interaction, most typically one-to-one conversations with the same stranger, is selected in order to activate the patient’s key social fears, while not being excessively anxiety provoking. The two interactions happen under two different conditions: first, while focusing attention on themselves, monitoring their performance (e.g. thinking how they are coming across to the other person) and using safety behaviours (such as preparing what to say); and second, while focusing externally, getting lost in the conversation (rather than evaluating themselves) and dropping safety behaviours (e.g. speaking spontaneously). The two conversations are video recorded for later potential video feedback.

#### The self-focused attention and safety behaviours experiment can be done remotely by recreating social interactions via the webcam that would activate the person’s key fears

For many patients we find having a short conversation with a stranger activates their social fears and sufficient anxiety to provide a helpful learning experience. In remote therapy this can be done by adding a colleague to the call. If this is not possible, the therapist could role-play the stranger. However, the preferred option is for a stranger to take part in the two conversations, as this provides a more meaningful social interaction with more convincing written feedback on the conversations. It also avoids the role of the therapist being confused with a phobic object. As in face-to-face therapy, the therapist would ideally have identified a person for the patient to speak to in advance and arranged for them to be free for sufficient time for both interactions. The therapist remains on the call during the two conversations. For a small number of patients with predominantly public speaking anxiety, speaking to a stranger may not activate their key social fears. In this case the patient may need to give two brief formal presentations by standing up in front of the webcam and presenting to the therapist and somebody else. The social task chosen should be driven by the patient’s social fears. For example, a patient who believed her hands shook if she held a cup while speaking had two brief social conversations, the first while she held the cup tightly and focused on herself and in the second she held the cup loosely and focused on the conversation.

As in face-to-face treatment, in order to ensure there is a notable difference in use of safety behaviours and internal self-focus between the two conditions, and to help patients compare their experience of the two different conversations, a number of 0–100% ratings are taken immediately after each interaction:

1. How much was your attention focused on yourself and how you were coming across?
2. How much were you using your safety behaviours?
3. How anxious did you feel?
4. How self-conscious did you feel?
5. How much did you think [patient’s specific fears] happened?
6. How did you think the conversation went overall?

**Figure 2.**
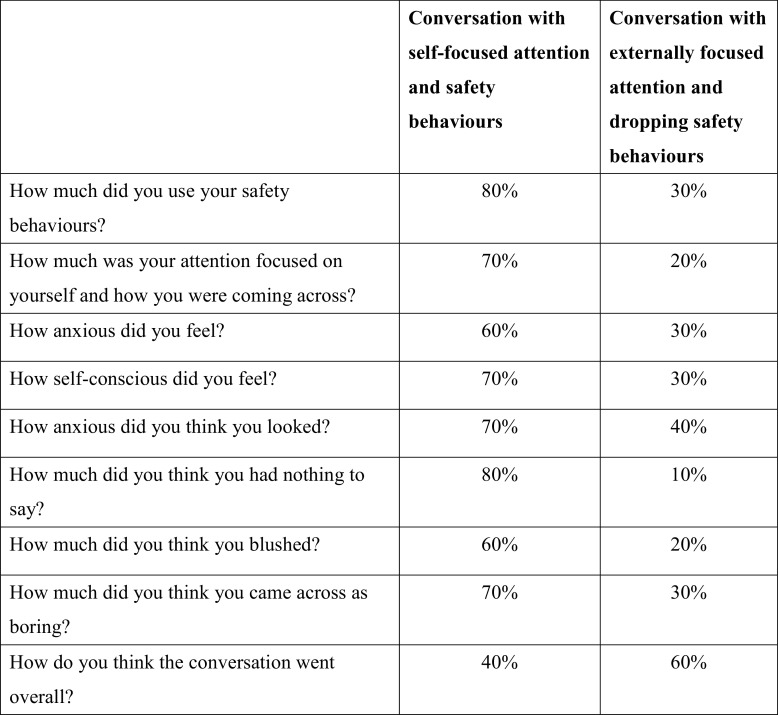
An example of a table that was completed and shared via screen share after the self-focused attention and safety behaviours experiment was carried out via video conferencing.

A two-column table, as shown in Fig. 2, is drawn up to facilitate comparison between the conversations and can be reviewed by sharing the screen. When reflecting on the conversations and looking with the therapist at this table, patients usually discover that focusing externally and dropping safety behaviours leads to them to feel less anxious and think they come across better. The therapist asks the person that the patient spoke to during the experiment to provide written feedback (e.g. via email) that will be used to further consolidate key lessons learnt from video feedback.

### Video and still photograph feedback

Video and still photograph feedback are used throughout therapy to update patients’ negative, distorted self-images. Video feedback is first typically used to view recordings of the self-focused attention and safety behaviours experiment carried out in the previous session. To help patients see the difference between their negative self-image and what is actually shown on video, careful attention is paid to setting up the video recording, preparing patients to view the video objectively, and subsequently watching and discussing the footage with the therapist.

#### When therapy is remote, patients and therapists watch recordings of experiments together on screen

**Figure 3.**
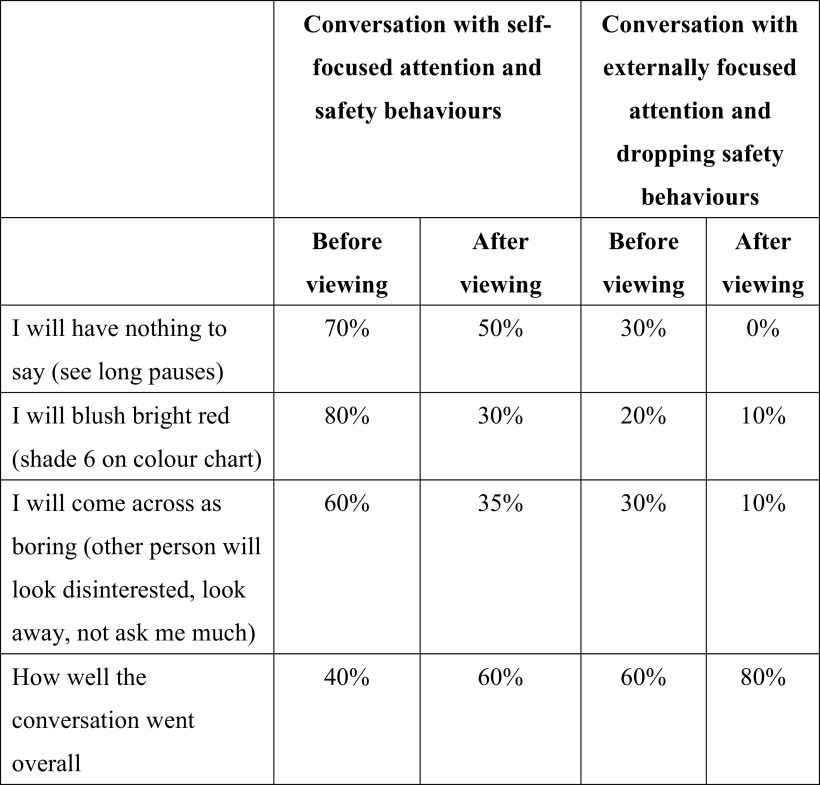
An example of a four-column table that was completed and shared via screen share when doing video feedback of the self-focused attention and safety behaviours experiment via video conferencing.

As in face-to-face therapy, patients are first asked to make observable predictions about how they think they will come across on video in both conversations (e.g. I will have nothing to say, we will see long pauses 80%). Patients are asked to demonstrate what they think their feared concerns will look like for later comparison with the video image (e.g. selecting a shade of red that they felt they blushed from a colour chart sourced from Google images). The therapist then guides the patient to ‘*watch the screen like you are watching a television show, as if you are watching two strangers having a conversation. Look at everybody in the conversation, not just one person*’. Therapist and patient then view the videos of each conversation together, re-rating the patient’s predictions after viewing each video and pausing and rewinding to discuss any moments of concern. A four-column table (see Fig. 3), is used to compare the patient’s ratings before and after viewing recordings of the two parts of the self-focused attention and safety behaviours experiment. This can be completed in a Word document and viewed together on screen. This usually helps patients to discover that they come across much better than predicted in both conversations, and possibly even better when dropping their self-focused attention and safety behaviours. It is a key opportunity for patients to find out that their thoughts, feelings and self-image are not a reliable guide of how they are coming across to others.

#### Pause video footage of the patient early on to check that their ‘inner critic’ or anxious feelings have not been re-activated whilst viewing

When doing video feedback therapists are encouraged to observe the patient’s facial and bodily reactions to the video to ensure that their ‘inner critic’ has not been activated. For example, a patient may cringe or recoil when being hard on themselves about their performance. A challenge of doing video feedback remotely, when the therapist is also watching the footage, is that it can be difficult to pick up on these subtle signs. Pausing early on when viewing the video to ask: ‘*Are you watching all the people on screen as if they are strangers?*’, ‘*What is your general impression of the chat so far?*’, will indicate whether patients have been watching themselves as if they were a stranger, without their anxious feelings being reactivated. For a detailed therapist guide on using video feedback in CT-SAD, including suggestions on addressing a range of potential processing biases, including self-criticism, see Warnock-Parkes *et al*. (2016).

#### Feedback from the conversational partner(s) can also be viewed together by screen share, or the feedback copied and emailed to the patient

If patients are highly self-critical, it can be helpful to share the conversational partner’s feedback before viewing the video, as this can help patients to view from the other person’s perspective, rather than through the eyes of their self-critic. It is recommended to read the conversational partner’s feedback first to ensure what has been written is helpful to share with for the patient (see OxCADAT resources site for a guide on using conversational partners and feedback).

#### Taking a screen shot of behavioural experiments recorded via the webcam can freeze key moments of belief disconfirmation in a photograph

For example, a patient felt like he said something stupid and had a frozen look on his face when speaking to a stranger via the webcam. When viewing this moment on the recording, his face did not look frozen and the other person did not appear to notice. The therapist captured this moment by taking a screenshot of the recording. These images can be turned into a digital or printed out flashcard by adding some text that highlights key learning points (e.g. ‘I look more acceptable than I feel – ignore my feelings’). See Warnock-Parkes *et al*. (2016) for printed examples.

### Attention training

The next step in CT-SAD involves helping patients to obtain more helpful information about how acceptable they are to others by carrying out behavioural experiments. During experiments patients need to switch their focus of attention externally onto the social interaction (e.g. focusing on the other person, being lost in the conversation rather than thinking about how they are coming across), in order to evaluate how others actually respond to them and whether or not their negative predictions happen. As self-focused attention has become automatic to many patients, formal training in shifting focus of attention before starting behavioural experiments is important. This is usually delivered in the session following video feedback. Attention training involves guiding patients through a number of exercises switching their focus of attention from internal (focused inwards on the self) to external auditory and visual stimuli (focused externally on the outside world).

#### All the attention training steps can be done remotely, by guiding patients to focus on sounds, colours, shadows or reflections in their home and then play a piece of music aloud

The exact order and type of exercises can be determined by the therapist. However, we usually introduce the following exercises, ending with the therapist reading to the patient, as this is most like a social interaction:

- Step 1: Sounds
- Step 2: Colours
- Step 3: Shadows/reflections or textures
- Step 4: Music
- Step 5: Therapist reading a passage of text

A range of attention training videos are available on the OxCADAT resources site. These videos involve a number of attention training exercises (outside scenes, people scenes, an auditory attention gym, a visual attention gym, crowd scenes and two extracts of books read aloud) and can be used in remotely delivered therapy when carrying out the above steps by screen-sharing the videos and completing the exercises together. We also provide a YouTube link that therapists can share with patients, giving access to a private channel hosting these videos so that patients can use them for ongoing homework practice.

#### Reducing self-consciousness during remote attention training

When attention training is done in face-to-face treatment, therapists make some adjustments to minimise patients’ self-consciousness during the exercises (such as adjusting the positioning of their chair so that they are not looking directly at the patient and shutting their own eyes when the patient is also instructed to do so, e.g. when listening to sounds or music). Therapists should do what they can to try similar manoeuvres to reduce patients’ self-consciousness in remote therapy. Viewing attention training videos together (see above) during a session of attention training practice can also help here.

### Behavioural experiments

From the exercises covered so far in therapy, patients have experientially discovered the unwanted effects of their self-focused attention and safety behaviours and learnt that the evidence they have been using to evaluate how they come across (their feelings, negative self-images and impressions) is misleading. The rest of therapy provides a systematic way of collaboratively generating more reliable evidence for how they actually come across, and behavioural experiments are a key vehicle for this. They are used in the majority of sessions and for homework from session 4 onwards. Experiments are collaboratively set up to test patients’ key social cognitions (e.g. I will shake, sweat, etc.), dysfunctional assumptions (e.g. I have to speak fluently to be accepted), unconditional beliefs (e.g. I am unlikeable, etc.) and negative self-images/self-impressions (e.g. I am red as a beetroot, I am different and apart from others). Referring back to the patient’s model and regular monitoring of questionnaires (such as the social cognitions questionnaire and the social attitudes questionnaire) is crucial in identifying the key beliefs and elements of the patient’s self-image/impression to target.

Early experiments start with dropping safety behaviours and externally focusing to discover what happens. As treatment progresses experiments move to discovering what happens if patients’ worst predictions were to occur – decatastrophising experiments. Here therapist and patient intentionally demonstrate the thing the patient is afraid of (such as saying something stupid or boring, or creating the appearance of a blush, shake or sweat) and then observe other peoples’ reactions.

#### All of the usual steps in setting up and carrying out experiments apply when delivering treatment remotely including:

1. *Identifying a key belief to test* (guided by the patient’s model and the most recent social cognitions questionnaires);
2. *Loosening the belief* (e.g. exploring what has already been learnt in treatment and the evidence for and against the belief, consider using surveys);
3. *Generating a situation to drop safety behaviours and test observable predictions* (e.g. ‘When speaking to a small group via the webcam, if I drop my safety behaviours and speak spontaneously they will think I am boring, 60%, and I will know this because they will roll their eyes, or leave the chat early’);
4. *The experiment is carried out while the patient drops their safety behaviours, remains externally focused and observes the reactions of the other people involved*;
5. *Outcome and learning are discussed and generalised* (the goal is to help patients update their negative self-imagery and discover that they are acceptable when they are just being themselves); and
6. *Further experiments are set up for homework*. Whenever possible, video, other person and still photograph feedback can be used when carrying out behavioural experiments.

#### Behavioural experiment record sheets are shared remotely and used to plan and record the outcome of experiments

**Figure 4.**
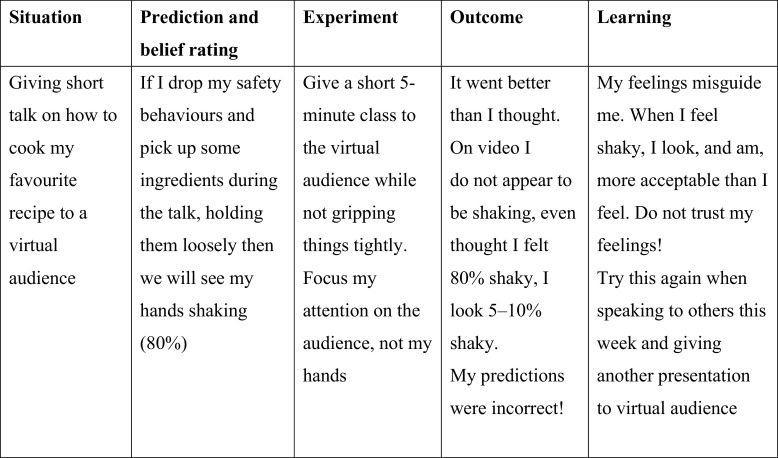
Example behavioural experiment record sheet completed by a patient carrying out a behavioural experiment giving a presentation to a virtual audience.

As much as possible the patient is encouraged to complete the record sheet themselves and screen share from their side of the call. An example of a completed behavioural experiment record sheet can be seen later in this clinical guide in Fig. 4.

#### A challenge to overcome in remotely delivered CT-SAD is that the therapist and patient cannot carry out behavioural experiments together in person

In face-to-face therapy some experiments are done in the therapy office, by inviting people into the room for the patient to speak to, and some by leaving the office together and experimenting in the outside world (e.g. speaking to strangers on the street, going into nearby shops, etc.). This poses a challenge for CT-SAD when delivered remotely. To get around this problem, therapists can invite colleagues to join webcam calls to take part in brief social interactions or role-plays (e.g. role playing a work meeting, college tutorial). Alternatively, patients could make a telephone call experiment during the video session with the therapist present, or be encouraged to leave their home^1^ to carry out an experiment in public with their therapist on the phone.

*Adam was worried he would freeze and sit silently if he did not prepare things to say. He called a local shop on his mobile phone during a video conference session to ask whether they were offering food deliveries. During the call he dropped his safety behaviours (preparing and rehearsing what to say) and focused on the other person rather than himself.*

*Marzena felt stared at when walking outside. During a session Marzena’s therapist suggested she go for a walk, while they continued to speak on the phone. Marzena was asked to look down, bring on the feeling of being stared at and make a prediction of how many people she felt were staring at her, before then looking up and around and observing what others were actually doing. She discovered that hardly anybody was looking at her.*

*Clare was worried that if she did not talk about interesting topics other people would find her boring and reject her. During a video conference call her therapist invited a colleague to join the call for Clare to carry out a decatastrophising experiment. During the chat Clare was encouraged to purposefully talk about topics she thought were mundane (e.g. household chores).*

#### The online world can provide a range of opportunities for behavioural experiments

Research suggests that people with social anxiety also experience anxiety and activation of their social cognitions (e.g. people think I am boring or stupid) during online interactions, such as posting or messaging on Facebook (Carruthers *et al*., 2019; Warnock-Parkes et al., in preparation). An interview study conducted with patients found that all elements of the cognitive model of SAD were present during interactions without the physical presence of another person, including negative self-images/impressions of patients’ online selves (‘I have the impression I look boring, like I don’t exist’). Patients also described using similar safety behaviours online to their face-to-face interactions (such as censoring posts, avoiding sharing anything about themselves, trying to make a good impression and rehearsing things to post) (Warnock-Parkes *et al*., in preparation). This shows that the online social world is not the safe haven some may expect for people with SAD, but it can provide a range of opportunities for behavioural experiments. This can be particularly helpful when opportunities for face-to-face social contact are limited, as has been the case during the COVID-19 pandemic with associated social distancing measures.

A range of ideas for experiments that can be carried out in remotely delivered treatment to test a range of beliefs are outlined in Table 3.

**Table 3.** Suggestions for behavioural experiments that can be done in remotely delivered therapy both in session and for homework

Appraisal	Example experiments	Carried out in session/homework
**I’m being stared at**	Walk to local park or high street with therapist on the telephone. Look down and bring on feeling of being stared at. Make prediction based on feelings then look up and observe how many people are actually staring	In session (repeat for homework)
	Draw attention to myself by dropping keys in public place with therapist on telephone and look around to see how many people stare (decatastrophising)	In session (repeat for homework)
**I’m boring**	Group conversation with two strangers on webcam. Speak spontaneously and focus on conversation rather than monitoring myself. Watch video afterwards and get feedback from the people I spoke to	In session
	Join group activity (e.g. online choir/course). Be myself rather than only trying to say witty or interesting things	Homework
	Reach out to a friend online and suggest a session of online gaming during which I will not rehearse interesting things to say but instead speak spontaneously	Homework
	During a meet up or WhatsApp chat purposefully say things I think are boring and see how my friend responds (decatastrophising)	Homework
**I’m stupid**	Conversation with stranger on webcam. Do not plan clever things to say	In session
	Message a colleague and do not prepare things I think sound intelligent to say, just write whatever comes to mind	In session
	Ask a question I think is stupid to customer services of an online store (decatastrophising)	Homework
	Say something to a friend on in person/on FaceTime I think is stupid (decatastrophising)	Homework
**I’ll have nothing to say**	Call an online store to enquire about a purchase (e.g. ask if I can send it back if it does not fit). Do not prepare what to say, speak spontaneously Purposefully pause during a group conversation on webcam Listen to others’ conversations over the week, notice any pauses and how people respond to these Virtual audience presentation, do not script, speak spontaneously and record doing this – watch video back Purposefully pause when speaking to a friend/calling a customer services helpline (decatastrophising)	In session In session Homework Homework
**I’ll blush**	Speak to a stranger via the webcam, observe reactions if I feel red. Watch video of conversation afterwards and compare how red I actually look to my predictions on a red colour chart	In session
	Put blusher on cheeks, walk to local shop and buy something, focusing externally (decatastrophising)	In session
	Put blusher on cheeks before work video conference call. Take a screen shot if possible (decatastrophising)	Homework
**I’m sweaty**	Do not wear my jacket during a group meeting role-play via webcam. Ignore how sweaty I feel and focus on the conversation	In session
	Give a short presentation to a stranger via the webcam with water on my armpits and forehead (decatastrophising)	In session
	Wear light coloured shirt to work meeting and speak up more, focusing on meeting, not how sweaty I feel	Homework
	Meet up with or FaceTime a friend with water on my armpits. If possible take a FaceTime screen shot to capture their reactions (decatastrophising)	Homework
**I’ll shake**	Role-play work meeting with stranger on webcam and drink water while holding glass loosely	In session
	Purposefully shake during a webcam chat with a stranger and observe their reactions. Watch video of the chat afterwards and compare how shaky I look to predictions based on feelings (decatastrophising)	In session
	Drink some tea during a meet up/Facetime with friends or colleagues and hold cup loosely	Homework
	Purposefully shake when speaking to my neighbour (decatastrophising)	Homework

### Surveys

Behavioural experiments are the best way of helping patients test out their negative beliefs and predictions about social interactions. For this reason, the core of all courses of CT-SAD needs to include multiple behavioural experiments. However, surveys can be a further helpful way to loosen negative beliefs (e.g. If I sweat people will think I’m incompetent) and to normalise many of the physiological sensations patients worry about. Some example survey questions to address the belief ‘people will think I am weird if I blush’ might include: *Do you blush? Have you ever noticed other people blushing? If you notice somebody blushing what do you think? How much would you think it meant the person blushing was weird 0–100%?* Therapists should read survey responses before sharing with patients to ensure that respondents have understood the questions and responded helpfully. In remote therapy survey responses can be shared by email or read together by sharing screen. There are some images of famous people sweating and blushing online that patients may find normalising to see. Some collated images are available on the OxCADAT resources website (oxcadatresources.com) that can be downloaded in PDF format and shared with patients during discussion to loosen their beliefs (e.g. *Do you think these people look anxious/incompetent*? *What do you make of that?*, etc.).

### Using virtual audiences

In face-to-face CT-SAD patients who struggle with public speaking or being the centre of attention can benefit from carrying out a behavioural experiment giving a presentation during a session to a small audience. Arranging for a live audience to take part in such an experiment can be practically challenging, even more so when working remotely. In these circumstances some patients might find it helpful to use virtual audiences to carry out behavioural experiments, and to practise giving a number of presentations to different audiences to build their confidence in public speaking. For this purpose, a sharable link to nine options of virtual audiences hosted on YouTube is available through the OxCADAT resources website. A brief training video for therapists on using virtual audiences is also available. These virtual audiences vary in size and level of challenge (standard *vs* challenging). In more challenging audiences, some members appear somewhat distracted (e.g. looking at their watch, etc.).

#### Guidance on using virtual audiences

When giving presentations to virtual audiences during a remote therapy session or for homework, patients are encouraged to stand up and maximise the audience to full-screen, as this can help the experience feel more authentic. Importantly patients are directed to drop their safety behaviours and focus on the audience rather than themselves. If the person is recording their presentation then they should keep any live video feed out of view so that they are not distracted by their own video image and can focus externally on the audience. When using video footage recorded for video feedback, we recommend that an image of the virtual audience remains open next to the video footage of the patient (or the audience could be played again alongside the presentation). This can help the patient watch their presentation from the perspective of the audience, rather than using their feelings to judge what they see.

*Hussain feared he appeared anxious, with shaky hands, when centre of attention and gripped things tightly to prevent shaking. He and his therapist planned an experiment* [see Fig. 4] *presenting to a virtual audience while focusing externally and holding some items loosely in his hands. Reviewing video recorded of his presentation he was able to see that he did not appear shaky on video*.

#### Virtual audiences can also be used to carry out decatastrophising experiments

For example, a patient who worried about freezing when centre of attention purposefully added a pause into her presentation, then viewed a recording of this, discovering it did not look as noticeable or catastrophic as it felt.

### Dealing with anticipatory worry and post-event rumination

Many patients with SAD worry in advance of social situations or ruminate on their performance after the event. Therapists and patients can keep track of these processes during therapy by using questionnaires (e.g. SPWSS, see Table 1). If no improvement in these processes is being observed, some patients may benefit from a focused intervention. Here we find helping patients to explore the advantages and disadvantages of anticipatory worry or post-event processing is helpful. Patients are encouraged to switch from worrying/dwelling to actively testing out their worries (‘how can I test this out using a behavioural experiment?’).

#### Post-event processing can be particularly problematic with social media use, as immediate feedback from others is often not possible

This can raise problems for patients carrying out behavioural experiments online (e.g. after posting something on Facebook or Twitter there may be some delay before anybody responds). Patients may benefit from a flashcard stuck to their computer, or a screensaver on their telephone to remind them to switch their focus of attention away from post-event rumination after using social media and instead to get absorbed in a different task.

### Updating negative self-images and impressions

#### Key to recovery in CT-SAD is ensuring that patients’ negative self-images and impressions are updated

Consideration of patients’ self-images/impressions needs to be included in behavioural experiments and discussed when generalising learning from experiments and video feedback (e.g. ‘*what does this tell you about the image you have of yourself as having frozen eyes like a rabbit in headlights?*’, ‘*From what we saw on video how do you actually come across?*’, ‘*Can we take a photo from the video as a reminder of your updated self-image?*’). For some patients their self-image is clearly linked to cognitions that are measured weekly on the social cognitions questionnaire (e.g. I am blushing, I am sweating) and change can be measured by reviewing their ratings on these items. Some patients have more idiosyncratic self-images/impressions that would not be included in the process questionnaires (e.g. seeing myself with a silly smirk, feeling detached and left out, looking like a small child). Therapists might find it helpful to ask patients to rate how much they believed they came across this way over the week (0–100%) and ‘*in social situations you found challenging this week what was your image or impression of how you came across?*’.

#### In a small number of cases we find that negative self-images/impressions persist, despite carrying out the interventions described

For these patients we would recommend exploring past experiences of social trauma that could be linked in meaning to the present day self-image/impression. For example, being ridiculed for blushing when reading aloud in class, linking to a current self-image of looking bright red or repeated school bullying, linking to a negative impression of the self as ‘inferior and vulnerable’ in the present day (Hackmann *et al*., 2000). For these patients, a session working on socially traumatic memories, usually later in therapy, can be helpful.

#### Techniques for working on socially traumatic memories

##### Discrimination training is usually employed first to help patients process current social situations without being haunted by past social traumas

Patients are encouraged to practise looking out for key differences between their socially traumatic memories and the present social situation (e.g. THEN I was 10 and at school, bullied by classmates. NOW I am 34, in a work meeting and not being bullied). When doing discrimination training remotely, screen share could be used to write out a table of differences between THEN and NOW when thinking about recent social situations when socially traumatic memories were activated. Patients might find it helpful to create a paper or digital flashcard of key differences that could be used as a reminder before entering present-day social situations. For remote interactions the flashcard could remain visible to the patient during the interaction (e.g. next to their laptop, device or telephone during a video call) as a reminder of the key differences between the past and the present situation.

##### Imagery re-scripting of socially traumatic memories may be needed for some patients

Discrimination training, followed by patients practising this in a range of social situations for homework, is usually sufficient in weakening the link between present day social situations and the socially traumatic memory, stopping re-activation of the memory in future. For some patients, memories will continue to intrude and imagery re-scripting could be helpful. The procedure used is outlined in detail by Wild and Clark (2011) and the key steps briefly summarised in Table 4.

**Table 4.** Summary of the key steps involved in imagery re-scripting for some patients with distressing socially traumatic memories

Step	Details of procedure
**1**	A key memory that links to the patient’s negative self-image or impression is identified
**2**	The negative meaning that encapsulates how the person sees themselves in present-day social situations is identified and rated (e.g. ‘I am dull and will be ridiculed by others again – 80%’)
**3**	Alternative evidence for this negative meaning is explored using Socratic questioning exploring the memory from an adult perspective, e.g. ‘*What do we know about this now, from the experiments you have done in treatment?*’, ‘*If a young girl told you that girls at her school treated her in this way, would you tell her that this meant that she was dull?*’, ‘*What would you say to her?*’
**4**	The new information discovered through cognitive restructuring is then brought back into the memory to update it through imagery re-scripting. Patients are instructed to picture the memory in their mind and talk through it in the present tense in three phases: (a) Through the eyes of their younger self, experiencing the event (b) Through the eyes of the adult self, observing what happened to their younger self and intervening, bringing in new information (c) Through the eyes of the younger self again, but this time experiencing the older self intervening, offering compassion and bringing in the new information to update the memory

##### A supportive therapeutic relationship facilitates successful remote delivery of imagery re-scripting

Increasing verbal empathy, warmth and encouragement when doing imagery re-scripting remotely can be helpful (e.g. ‘You are doing really well’, ‘That sounds really difficult, well done for talking through it’). Usually after successful imagery re-scripting patients report a shift in affect and that the negative meaning associated with their early memories no longer feels as true.

##### Increase verbal checks on affect when doing imagery re-scripting remotely

It may be harder to detect subtle facial and bodily cues of emotional distress or avoidance (e.g. looking at other applications) when doing imagery re-scripting remotely. Therefore, therapists may want to do more verbal checks with the patient to ensure an optimal level of emotional engagement during re-scripting: ‘*You are doing really well, how distressing is it to talk through the memory in this way?*’.

*Patients may benefit from having access to items at home that show them how different things are now compared with their memory* (e.g. recent photos of themselves as an adult with friends showing they are no longer being bullied) that they could use during memory re-scripting as a reminder that the memory is in the past and the present day is different.

### Addressing unconditional negative beliefs about the self and self-criticism

#### Unconditional negative beliefs about the self are common in SAD and need to be targeted in behavioural experiments throughout therapy

These beliefs (e.g. ‘I am unlikeable’, ‘I am incapable’, ‘I am vulnerable’, ‘I am inferior’) have often developed after many years of the limiting impact SAD has had on patients’ lives (e.g. reduced social network) and early social trauma (e.g. bullying, racism, etc.). In CT-SAD unconditional beliefs are targeted throughout therapy, and as early as session 2, by including them as predictions in the self-focused attention and safety behaviours experiment, video feedback and a range of behavioural experiments. Decatastrophising experiments are particularly important here, demonstrating to the patient that even when they show perceived ‘flaws’ (e.g. making mistakes, blushing, sweating, etc.) they are acceptable; these are just part of being human. Taking time to help patients generalise their learning from these therapy tasks is essential, e.g. ‘*what does this experiment/the feedback you just read/what we saw on video tell us more generally about the belief you are unlikeable?*’. Patients typically notice significant improvement in their unconditional beliefs after behavioural experiments showing they are more acceptable, likeable and capable than they feel. As patients start to achieve their therapy goals (e.g. speaking up in a work meeting, socialising more with friends), further improvement in unconditional beliefs is usually observed.

#### On occasions unconditional beliefs may persist, despite carrying out the interventions described

In these cases, working on experiences of early social trauma with linked cognitive meanings to the unconditional belief (e.g. bullying at school which the patient interpreted to mean they were unlikeable) may be helpful. Patients may also keep a positive data log (Padesky, 1994) alongside their treatment, recording any evidence, however small, which fits with an alternative self-belief they would like to develop (e.g. I’m acceptable). Habitual-criticism usually reduces using the standard CT-SAD interventions described. However, if this remains high as therapy progresses it can be a blocking process for some patients, limiting generalised learning from behavioural experiments and maintaining unconditional beliefs. In these cases helping patients to spot and label their habitual self-critic, reminding themselves that ‘*that’s my internal bully, not fact and not what anybody else thinks*’, and responding in the more compassionate way they would to a friend can also be helpful. A flashcard can be useful here. An advantage of remotely delivered therapy is that patients are already in their home environment, so can be encouraged get up during the session to stick a flashcard somewhere noticeable (e.g. kitchen fridge) or leave their positive data log in place where they plan to add to it later that evening (e.g. bedside table).

### Therapy blueprint

The blueprint is a document collaboratively developed by the patient and therapist towards the end of therapy that summarises the key learning from treatment, steps to build on this learning and how to manage any future setbacks. For remotely delivered therapy, the blueprint can be emailed to patients to complete for homework and reviewed together by sharing screen. Additional content can be added as required. Patients should be encouraged to save their blueprint in an easily accessible place so that it can be retrieved as needed in future.

## Footnotes

1 At the time of writing, social distancing is mandated outside the home and all suggested experiments should be compliant with government guidance.

## Key practice points

(1) All of the core interventions of CT-SAD can be effectively delivered remotely via video conference with some adaptations.
(2) Remotely delivered therapy might be particularly appealing to patients with SAD who often avoid help-seeking due to anxiety.
(3) During remote sessions therapists may want to reduce intense eye contact early in treatment and ask for feedback regularly, as there are fewer visual cues.
(4) Behavioural experiments can be carried out during remotely delivered therapy sessions in a number of ways (e.g. the patient speaking to strangers via the webcam or on the telephone, leaving home to do an experiment in public with the therapist on a mobile telephone, or using virtual audiences).
